# Monocyte Distribution Width as a predictor of community acquired sepsis in patients prospectively enrolled at the Emergency Department

**DOI:** 10.1186/s12879-022-07803-7

**Published:** 2022-11-14

**Authors:** Ennio Polilli, Giancarlo Di Iorio, Claudio Silveri, Gilda Angelini, Maria Chiara Anelli, Jessica Elisabetta Esposito, Milena D’Amato, Giustino Parruti, Fabrizio Carinci

**Affiliations:** 1 Clinical Pathology Unit, Pescara General Hospital, Pescara, Italy; 2 Emergency Department, Pescara General Hospital, Pescara, Italy; 3 Beckman Coulter Srl, Milan, Italy; 4 Department of Biotechnological and Applied Clinical Sciences, University of L’Aquila, L’Aquila, Italy; 5 Infectious Diseases Unit, Pescara General Hospital, Pescara, Italy; 6 Department of Statistical Sciences, Bologna University, Bologna, Italy

**Keywords:** Early diagnosis, Emergency Department, MDW, Sepsis, Infection

## Abstract

**Background:**

Monocyte Distribution Width (MDW), a simple cellular marker of innate monocyte activation, can be used for the early recognition of sepsis. We performed an observational prospective monocentric study to assess the predictive role of MDW in detecting sepsis in a sample of consecutive patients presenting at the Emergency Department.

**Methods:**

Prospective observational study using demographic and clinical characteristics, past medical history and other laboratory measurements to predict confirmed sepsis using multivariate logistic regression.

**Results:**

A total of 2724 patients were included in the study, of which 272 (10%) had sepsis or septic shock. After adjusting for known and potential risk factors, logistic regression found the following independent predictors of sepsis: SIRS equal to 1 (OR: 2.32, 1.16–4.89) and 2 or more (OR: 27.8, 14.8–56.4), MDW > 22 (OR: 3.73, 2.46–5.70), smoking (OR: 3.0, 1.22–7.31), end stage renal function (OR: 2.3, 1.25–4.22), neurodegenerative disease (OR: 2.2, 1.31–3.68), Neutrophils ≥ 8.9 × 10^3^/µL (OR: 2.73, 1.82–4.11), Lymphocytes < 1.3 × 10^3^/µL (OR: 1.72, 1.17–2.53) and CRP ≥ 19.1 mg/L (OR: 2.57, 1.63–4.08). A risk score derived from predictive models achieved high accuracy by using an optimal threshold (AUC: 95%; 93–97%).

**Conclusions:**

The study suggests that incorporating MDW in the clinical decision process may improve the early identification of sepsis, with minimal additional effort on the standard procedures adopted during emergency care.

## Background

Sepsis is a clinical syndrome caused by dysregulated host response to infections occurring in approximately one third of hospitalizations [1–3], frequently leading to multiple organ dysfunction syndrome and death [4, 5]. As a life threatening condition [6], it needs to be properly tackled through targeted actions addressed by the Surviving Sepsis Campaign (SSC), including fluid resuscitation, broad-spectrum antibiotics within one hour from diagnosis, blood cultures and targeted antibiotic therapy [3]. These measures can effectively reduce mortality for all sepsis-related conditions [7–9].

Since the majority of cases may have already developed sepsis before hospitalization [10], the early assessment of subjects at presentation to the Emergency Department (ED) has been a matter of increasing interest for the identification of effective medical interventions [11–13].

The clinical confirmation requires a multidimensional assessment of different aspects e.g. individual risk factors, the clinical suspicion of infection and the possible organ dysfunction indicated by radiological examination and laboratory tests [1, 11]. However, the early recognition of sepsis is still hampered by ambiguous clinical signs and the limited accuracy of laboratory biomarkers e.g. the C-Reactive Protein, Procalcitonin and Lactic acid [11, 12, 14, 15].

As a result, there is currently no established gold standard for the early identification of sepsis that can drive the timely administration of therapeutic agents effectively. In routine emergency care, it is still difficult to discriminate between infectious vs non-infectious cause of illness, bacterial vs viral etiology and sepsis vs uncomplicated infections [16].

Moreover, the existing strategies are complex to apply within the difficult operating conditions of the ED, where patients with complex clinical conditions show up simultaneously with heterogeneous symptoms and rapid diagnostic testing may not be available, due to the high workload and the tight time constraints.

The present study aimed to improve the early identification of sepsis, by building a prediction model including Monocyte Distribution Width (MDW) in addition to other known predictors, using a large sample of consecutive patients admitted at the ED of a regional hospital.

## Methods

### Study population

The study population included consecutive patients aged ≥ 18 years with an available Complete Blood Count (CBC) analysis, admitted for any reason to the ED of the Pescara General Hospital (PGH), a large reference regional hospital located in the Abruzzo Region, Central Italy, between July 15th, 2020 and September 15th, 2020. Patients underwent CBC analysis and other laboratory parameters within < 12 h after ED presentation. They were excluded if either discharged from ED within 12 h, pregnant, leaving the ED before discharge, with incomplete clinical or laboratory data, or with missing MDW after CBC analysis (corresponding to patients with specific blood conditions e.g. immunocompromised, oncoematological, etc.).

The study was carried out in accordance with the amended Declaration of Helsinki. The study was approved by the local Ethics Committee (“Ethics Committee of Chieti—Pescara for biomedical research”, approval no. 539, May 2020). All patients provided their written informed consent to use anonymised clinical and laboratory data for institutional research purposes upon admission to the ED.

### Clinical data

The following personal characteristics were accurately recorded at presentation to the ED: demographic and clinical characteristics including age and gender, vital signs (heart rate, respiratory rate, body temperature, blood pressure), and history of comorbid conditions. We also measured comorbidities involved with organ dysfunction and sepsis [17, 18]: hypertension, dyslipidemia, diabetes, obesity, smoke, drug addition, anemia, acute myocardial infarction, transient ischemic attack, other cardiovascular diseases, chronic obstructive pulmonary disease, endocrinologic diseases, end-stage renal disease, hepatitis, connective tissue diseases, other immunological diseases, neurodegenerative diseases, other rheumatological diseases, metabolic diseases, bacterial infections, and underlying conditions potentially associated with a dysregulation of the immune system e.g. HIV infection, malignancy, hematologic diseases.

### Laboratory assays

All patients admitted to the ED underwent blood testing for the following parameters: CBC, C-Reactive Protein (CRP) and Procalcitonin (PCT) when available. CBC and MDW determinations were analysed using the hematologic analyzer UniCel DxH 900 (Beckman Coulter, Inc, Brea, California). All determinations of MDW were measured from a K3EDTA whole-blood venous sample within 2 h of collection and were performed in the same blood tubes used for CBC determinations. Turnaround Time for such measurements was within the time of CBC. Quality control of CBC including MDW were performed daily with COULTER^®^ 6C Plus Cell Control and COULTER LATRON CP-X Control, a suspension of stable polystyrene particles of uniform size. Latron CP-X was used as part of the daily quality control procedure, to monitor the stability of the electrical processing and the fluidic flow rate systems used to measure the volume, conductivity and light scattering characteristics of cells as they pass through the flow cell. COULTER^®^ 6C Plus Cell Control was used by monitoring performances of diagnostic processes. Controls with known characteristics were analysed daily in the same way as samples, while results of the analysed controls were compared with standard characteristics using statistical methods calculated by the same instrument [19]. Producers of hematological analysers did not provide any unit for MDW [20, 21]. CRP was measured using the chemoluminescence method (Abbott Diagnostics, Lake Forest, USA). All results of blood cultures processed by the local laboratory of Microbiology were also included.

### Outcome definition

Sepsis-2 was applied to screen sepsis at admission to the ED [22]. Patients with sepsis were classified as either non-SIRS or controls (SIRS eq 0,1 and no infection), infection (SIRS eq 0,1 and suspect/confirmed infection), SIRS (SIRS ≥ 2 and no infection), or sepsis (SIRS ≥ 2 and infection). Infection was identified on the basis of clinical features, imaging studies and microbiological findings available for each patient. In the absence of any infection work-up performed within 12 h of the initial CBC, patients were considered not infected. In addition, as infection or sepsis may be evident after the initial work-up in the ED, data collection and clinical and laboratory results were retrospectively reviewed by trained investigators to evaluate clinical features, radiological findings and microbiological results up to 24 h after the initial laboratory analyses. These findings were used to revise diagnoses considered uncertain or possibly inaccurate due to incomplete information, and finally classify the patient. Patients classified as sepsis were further categorized as septic shock when presenting also a diagnosis of refractory hypotension.

### Statistical analysis

Descriptive analysis included the calculation of the mean (standard deviation) or median (interquartile range) for continuous variables, and absolute and relative frequencies for categorical variables. Significance levels and 95% confidence intervals (95%CI) were based on alpha = 0.05. The differences between patients with or without confirmed sepsis were tested using chi-square for categorical variables [23].

The reliability of MDW in predicting sepsis was investigated through ROC analysis, including the calculation of Sensitivity, Specificity, Positive Predictive Value (PPV), Negative Predictive Value (NPV) and the Area Under the Curve (AUC).

Optimal cutoffs were used to define binary variables for MDW, WBCs, CRP, Neutrophils, Lymphocytes and Platelets to support clinical interpretation of univariate and multivariate logistic regression in terms of odds ratios (ORs) of sepsis [24]. An alpha level of 0.05 was used to present ORs together with their 95% confidence intervals and p values.

The association between the above binary predictors and sepsis was measured using adjusted ORs with 95% confidence intervals, taking into account all relevant demographic and clinical characteristics available through multivariate logistic regression [24, 25]. A total of 31 factors were included in a fully automated four-step backward elimination process, with age and gender forced in at all steps, and the other 29 variables sequentially screened for exclusion in three consecutive rounds, based upon p values greater or equal to 0.20, 0.10 and 0.05 respectively. The significance of the final model was measured with the likelihood ratio (LR) test, comparing the full model vs the model with the intercept alone, while the overall fit was checked using the Hosmer–Lemeshow (H–L) test [25]*.* Model accuracy was estimated using tenfold cross-validation, repeated 10 times.

Predictive risk scores for each patient in the database were computed as a weighted sum of all significant factors identified by multivariate regression, using coefficients corresponding to the increased risk calculated by rounding odds ratios to their next integer value [26]. We compared this empiric approach against the correct algebraic form derived from logistic regression [27].

A ROC analysis was performed using a separate 2 × 2 “confusion matrix” for every possible threshold applied to the total score [28]. The best threshold was defined as the one with the highest value of the Youden Index [29]. Point estimates and confidence intervals were computed using the bootstrap for sensitivity, specificity, Positive Predictive Value (PPV), Negative Predictive Value (NPV) and the De Long estimate of the 95% confidence interval of the Area Under the Curve (AUC) [25].

All analyses were performed using the R statistical language [30].

## Results

A total of 2896 consecutive patients presenting at the ED of the Pescara hospital were enrolled in the study during the reference timeframe. Among them, a total of N = 172 were excluded based upon the pre-defined exclusion criteria: N = 24 pregnant women, N = 94 subjects with inadequate sample collection or screening errors and N = 54 for whom MDW was not available.

**Fig. 1:**
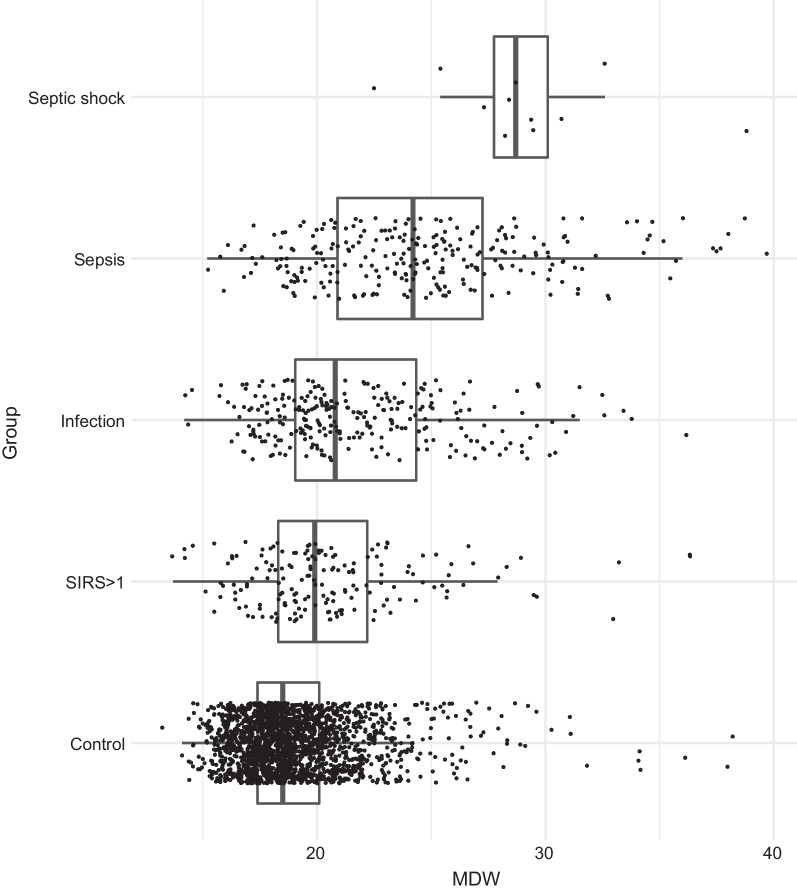
Variability of MDW by infection status

The results were based on a total sample of 2724 patients entering the final dataset. Among them, N = 272 (10%) had sepsis or septic shock. Higher median MDW values (Fig. 1) were found in sepsis (25.2) and septic shock (30.7), as opposed to infected (22.0), SIRS (21.2) and not infected (19.0).

A value of 22.0 was found to be the optimal cut point for MDW, while other laboratory biomarkers were dichotomized according to the ROC curve analysis: 12.2 × 10^3^/µL for WBCs; 8.9 × 10^3^/µL for Neutrophils; 1.3 × 10^3^/µL for Lymphocytes; 157 × 10^3^/µL for Platelet and 19.1  mg/L for CRP.

The distribution of the general characteristics and univariate odds ratios for sepsis are shown in Table 1. Among clinical variables, the following were significantly associated with sepsis: ERSD, neurodegenerative disorder and metabolic diseases. Among laboratory parameters, the strongest association was found for patients with CRP ≥ 19.1  mg/L and MDW > 22. Increased risk was also found for subjects with WBC ≥ 12.2 × 10^3^/µL, Neutrophils ≥ 8.9 × 10^3^/µL, Lymphocytes < 1.3 × 10^3^/µL and Platelet < 157 × 10^3^/µL.

**Table 1:** Clinical, laboratory variables and underlying health condition of study patients*

	Category	Overall	Sepsis		OR (95%C.I.)	p > χ^2^
			No	Yes		
Age**	Continuous		62.0 (20.6)	71.5(18.5)	1.03 (1.02–1.03)	< 0.001
Gender	Female	1314 (48.24)	1197 (48.8)	117 (43.0)	1.00 (–)	
	Male	1410 (51.76)	1255 (51.2)	155 (57.0)	1.26 (0.98–1.63)	0.069
Hypertension	No	1814 (66.59)	1651 (67.3)	163 (59.9)	1.00 (–)	
	Yes	910 (33.41)	801 (32.7)	109 (40.1)	1.38 (1.07–1.78)	0.015
Dyslipidemia	No	2471 (90.71)	2220 (90.5)	251 (92.3)	1.00 (–)	
	Yes	253 (9.29)	232 (9.5)	21 (7.7)	0.80 (0.50–1.27)	0.336
Diabetes	No	2380 (87.37)	2162 (88.2)	218 (80.1)	1.00 (–)	
	Yes	344 (12.63)	290 (11.8)	54 (19.9)	1.85 (1.34–2.55)	< 0.001
Obesity	No	2684 (98.53)	2418 (98.6)	266 (97.8)	1.00 (–)	
	Yes	40 (1.47)	34 (1.4)	6 (2.2)	1.60 (0.67–3.86)	0.317
Smoke	No	2624 (96.33)	2367 (96.5)	257 (94.5)	1.00 (–)	
	Yes	100 (3.67)	85 (3.5)	15 (5.5)	1.63 (0.92–2.86)	0.109
Drug addiction/alcoholism	No	2679 (98.35)	2414 (98.5)	265 (97.4)	1.00 (–)	
	Yes	45 (1.65)	38 (1.5)	7 (2.6)	1.68 (0.74–3.80)	0.241
Anemia	No	2584 (94.86)	2335 (95.2)	249 (91.5)	1.00 (–)	
	Yes	140 (5.14)	117 (4.8)	23 (8.5)	1.84 (1.16–2.94)	0.015
AMI	No	2483 (91.15)	2236 (91.2)	247 (90.8)	1.00 (–)	
	Yes	240 (8.81)	215 (8.8)	25 (9.2)	1.05 (0.68–1.63)	0.818
	Missing	1 (0.04)	1 (0.0)	0 (0.0)		
TIA	No	2011 (73.83)	1828 (74.6)	183 (67.3)	1.00 (–)	
	Yes	713 (26.17)	624 (25.4)	89 (32.7)	1.42 (1.09–1.86)	0.011
Other CVD	No	2568 (94.27)	2316 (94.5)	252 (92.6)	1.00 (–)	
	Yes	156 (5.73)	136 (5.5)	20 (7.4)	1.35 (0.83–2.20)	0.241
COPD	No	2521 (92.55)	2278 (92.9)	243 (89.3)	1.00 (–)	
	Yes	203 (7.45)	174 (7.1)	29 (10.7)	1.56 (1.03–2.37)	0.043
Endocrinological diseases	No	2567 (94.24)	2305 (94.0)	262 (96.3)	1.00 (–)	
	Yes	157 (5.76)	147 (6.0)	10 (3.7)	0.60 (0.31–1.15)	0.099
ESRD	No	2566 (94.2)	2339 (95.4)	227 (83.5)	1.00 (–)	
	Yes	158 (5.8)	113 (4.6)	45 (16.5)	4.10 (2.83–5.95)	< 0.001
Hepatitis	No	2678 (98.31)	2410 (98.3)	268 (98.5)	1.00 (–)	
	Yes	45 (1.65)	41 (1.7)	4 (1.5)	0.88 (0.31–2.47)	0.801
	Missing	1 (0.04)	1 (0.0)	0 (0.0)		
Connectivitis	No	2714 (99.63)	2443 (99.6)	271 (99.6)	1.00 (–)	
	Yes	10 (0.37)	9 (0.4)	1 (0.4)	1.00 (0.13–7.94)	0.999
Other Immune Disorder	No	2646 (97.14)	2380 (97.1)	266 (97.8)	1.00 (–)	
	Yes	78 (2.86)	72 (2.9)	6 (2.2)	0.75 (0.32–1.73)	0.477
Solid Tumor Malignancies	No	2421 (88.88)	2197 (89.6)	224 (82.4)	1.00 (–)	
	Yes	303 (11.12)	255 (10.4)	48 (17.6)	1.85 (1.32–2.59)	< 0.001
Hematologic malignancies	No	2660 (97.65)	2396 (97.7)	264 (97.1)	1.00 (–)	
	Yes	64 (2.35)	56 (2.3)	8 (2.9)	1.30 (0.61–2.75)	0.511
HIV infection	No	2720 (99.85)	2448 (99.8)	272 (100.0)	1.00 (–)	
	Yes	4 (0.15)	4 (0.2)	0 (0.0)	1.00 (0.05–18.59)	0.359
Neurodegenerative diseases	No	2464 (90.46)	2255 (92.0)	209 (76.8)	1.00 (–)	
	Yes	260 (9.54)	197 (8.0)	63 (23.2)	3.45 (2.51–4.74)	< 0.001
Metabolic diseases	No	2700 (99.12)	2434 (99.3)	266 (97.8)	1.00 (–)	
	Yes	24 (0.88)	18 (0.7)	6 (2.2)	3.05 (1.20–7.75)	0.034
SIRS	0	12 (0.44)	1405 (57.3)	13 (4.8)	1.00 (–)	
	1	862 (31.64)	825 (33.6)	37 (13.6)	4.85 ( 2.56- 9.17)	< 0.001
	2	333 (12.22)	184 (7.5)	149 (54.8)	87.52 (48.65- 157.45)	< 0.001
	3	98 (3.6)	35 (1.4)	63 (23.2)	194.54 (98.08- 385.85)	< 0.001
	4	13 (0.48)	3 (0.1)	10 (3.7)	360.26 (88.75–1462.44)	< 0.001
MDW	< 22	2176 (79.88)	2095 (85.4)	81 (29.8)	1.00 (–)	
	≥ 22	548 (20.12)	357 (14.6)	191 (70.2)	13.84 (10.43–18.37)	< 0.001
WBCs	< 12.2 × 10^3^/µL	2122 (77.9)	2025 (82.6)	97 (35.7)	1.00 (–)	
	≥ 12.2 × 10^3^/µL	602 (22.1)	427 (17.4)	175 (64.3)	8.56 (6.54–11.20)	< 0.001
Neutrophils	< 8.9 × 10^3^/µL	2013 (73.9)	1938 (79.0)	75 (27.6)	1.00 (–)	
	≥ 8.9 × 10^3^/µL	711 (26.1)	514 (21.0)	197 (72.4)	9.90 (7.46–13.15)	< 0.001
Lymphocytes	< 1.3 × 10^3^/µL ≥ 1.3 × 103/µL	1768 (64.9)	1676 (68.4)	92 (33.8)	4.23 (3.24–5.51)	< 0.001
	≥ 1.3 × 10^3^/µL	956 (35.1)	776 (31.6)	180 (66.2)	1.00 (–)	
Platelet	< 157 × 10^3^/µL	2360 (86.64)	2153 (87.8)	207 (76.1)	2.26 (1.67–3.06)	< 0.001
	≥ 157 × 10^3^/µL	364 (13.36)	299 (12.2)	65 (23.9)	1.00 (–)	
CRP	< 19.1 mg/L	1645 (60.39)	1601 (65.3)	44 (16.2)	1.00 (–)	
	≥ 19.1 mg/L	734 (26.95)	530 (21.6)	204 (75.0)	14.01 (9.97–19.68)	< 0.001
	Missing	345 (12.67)	321 (13.1)	24 (8.8)		

*Numbers in table are N, %, except for ** Mean, St.Dev

*AMI* Acute Myocardial Infarction, *TIA* Transient Ischemic Attack, *CVDs* Cardiovascular Diseases, *COPD* Chronic Obstructive Pulmonary Disease, *CRP* C-Reactive Protein, *WBCs* White Blood Cells, *ESRD* End-Stage Renal Disease

The results of multivariate analysis are shown in Table 2. The highest risk was found for patients with SIRS ≥ 2, which was over 27-fold compared to those non-SIRS, as opposed to those with SIRS = 1, who had just over twice the risk. Increased risk was also found for subjects with MDW > 22, smokers, patients with end stage renal function, neurodegenerative disorder, Neutrophils ≥ 8.9 × 10^3^/µL, Lymphocytes < 1.3 × 10^3^/µL and CRP ≥ 19.1 mg/L. The final model was significant according to the LR test (p < 0.001), while the H–L test did not show any evidence of poor fit (p = 0.46). The accuracy of the model estimated via cross-validation was equal to 0.927 (95%CI: 0.901–0.955).

**Table 2:** Logistic Multivariate Analysis for the prediction of sepsis*

Variable	β	(95%CI)		p > χ^2^	OR	(95%C.I.)	
		Lower limit	Upper limit			Lower limit	Upper limit
Constant term	− 6.3165	− 7.3127	− 5.4003	0.00	0.00	0.00	0.00
Age	0.0061	− 0.0041	0.0163	0.24	1.01	1.00	1.02
Gender	− 0.0034	− 0.3829	0.3762	0.99	1.00	0.68	1.46
Smoking	1.1025	0.1978	1.9895	0.02	3.01	1.22	7.31
ESRD	0.8338	0.2239	1.4390	< 0.01	2.30	1.25	4.22
Neurodegenerative Disorders	0.7873	0.2707	1.3024	< 0.01	2.20	1.31	3.68
SIRS = 1	0.8404	0.1521	1.5867	0.020	2.32	1.16	4.89
SIRS > 1	3.3242	2.6969	4.0317	< 0.001	27.78	14.83	56.35
MDW ≥ 22	1.3161	0.8990	1.7399	< 0.001	3.73	2.46	5.70
Neutrophils ≥ 8.9 × 10^3^/µL	1.0027	0.5976	1.4138	< 0.001	2.73	1.82	4.11
Lymphocytes < 1.3 × 10^3^/µL	0.5396	0.1538	0.9283	< 0.01	1.72	1.17	2.53
CRP ≥ 19.1 mg/L	0.9441	0.4898	1.4061	< 0.01	2.57	1.63	4.08

Significance of the model (Likelihood Ratio test): χ^2^ = 821.29, df = 11, p-value < 0.001

Overall fit statistics (Hosmer and Lemeshow goodness of fit (GOF) test): χ^2^ = 7.7745, df = 8, p-value = 0.46

*S.E. Standard Error, CI Confidence Interval, OR Odds Ratio. *ESRD* End-Stage Renal Disease, *SIRS* Systemic Inflammatory Response Syndrome, *MDW* Monocyte Distribution Width, *CRP* C-Reactive Protein

**Fig. 2:**
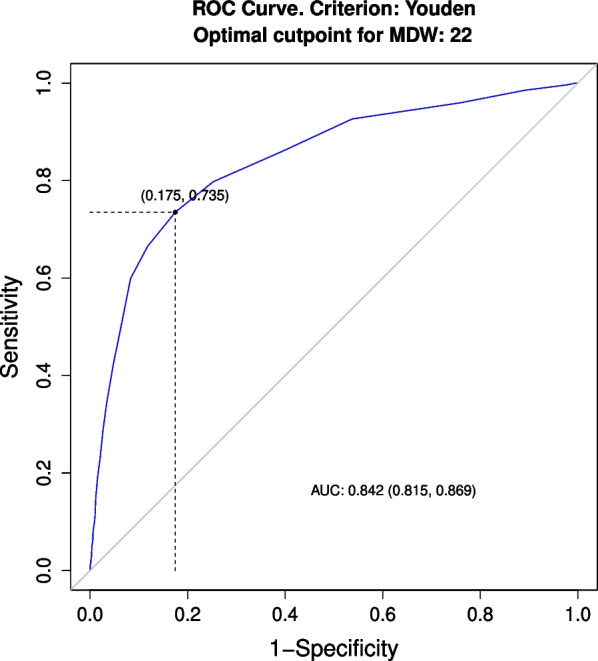
AUC and optimal cut point of MDW for sepsis prediction

The ROC curve for MDW to predict sepsis (see Fig. 2) showed a sensitivity level at the optimal cutoff of MDW > 22 equal to 74% (95%CI: 68–79%), with specificity = 83% (81–84%), PPV = 32% (30–38%) and NPV = 97% (96–97%).

The algorithm for the calculation of individual risk scores of sepsis is presented in Table 3. Using an optimal cut point equal to 11.5 achieved sensitivity of 91% (95%CI: 87–94%) and specificity of 88% (87–88%). PPV and NPV were respectively of 47% (42–51%) and 99% (98–99%). The overall performance was quite high (AUC = 95%; 93–97%), suggesting that the predictive model may be suitable for regular use at ED. The results obtained using the correct algebraic formula were practically identical.

**Table 3:** Algorithm for the calculation of the risk score

	**Sepsis**
Algorithm Condition value: [1 = Yes; 0 = No]	3* [Smoking] + 2* [Renal Failure] + 2* [Neurodegenerative Disorders] + 2* [SIRS = 1] + 28* [SIRS > 1] + 4* [MDW ≥ 22] + 3* [Neutrophils ≥ 8.9 × 10^3^/µL] + 2* [Lymphocytes < 1.3 × 10^3^/µL] + 3* [CRP ≥ 19.1 mg/L]
Optimal Cutpoint	** ≥ 11.5**

*SIRS* Systemic Inflammatory Response Syndrome, *MDW* Monocyte Distribution Width, *CRP* C-Reactive Protein

## Discussion

In this study, we examined the role of MDW as a laboratory marker of sepsis, in combination with other clinical variables measured in patients admitted to the emergency department (ED).

We found that a threshold for MDW ≥ 22 can be used as a laboratory biomarker showing an almost four-fold increased risk of sepsis, with a NPV equal to 97% that supports its use to rule out sepsis at presentation. Consistently with the results of recent literature [31, 32], our data suggest that other measures could be used for the early diagnosis of sepsis, independently from age and gender: smoking status, neurodegenerative disease, lymphocytes < 1.3 × 10^3^/µL, neutrophils ≥ 8.9 × 10^3^/µL, MDW ≥ 22, SIRS = 1, SIRS ≥ 2, and ESRD.

Different clinical explanations support the role of MDW as a predictive factor for sepsis. From a biological standpoint, monocytes are immune cells of the first phase of inflammation. Before involving adaptive immunity, they become activated by changing their morphology and increasing their volume, expressing new surface proteins e.g. CD16 [33, 34]. MDW is related to monocytes activation as a measure of diversity in the size of the cellular volume. For this reason, MDW reflects the early activation of the innate immune system during inflammation [14] and consequently can be slightly elevated in inflammation compared to normal conditions.

Few studies have investigated the potential association between MDW and sepsis using multivariate adjustment [35–37]. Recent papers focused on improving sepsis prediction through the combined use of MDW and other laboratory parameters. Crouser et al. (2019) demonstrated that the association of MDW with WBC improved the AUC for sepsis detection [22]. This result was recently confirmed [38], but did not show significant improvement using both PCT and MDW. Other reports addressed the use of CRP and MDW for the prediction of sepsis, without showing increased accuracy [38, 39].

Unfortunately, no consistent clinical manifestations or diagnostic indicators have been confirmed as a gold standard to recognize sepsis. Different sepsis definitions have been adopted over time to simplify its diagnosis, particularly during the phase of triage, where timely medical decision is required. Generally, these criteria combine physiological parameters of inflammation and organ dysfunction with the suspicion of infection. Inevitably, these methods often lead to diagnostic uncertainty, particularly in patients with vital signs or laboratory abnormalities that are not due to infections [40]. The third consensus of sepsis (Sepsis-3) recently introduced qSOFA as the standard criteria to recognize sepsis outside the ICU [2], whose performance in predicting sepsis at presentation to the ED showed to be poor. A recent paper reported that between 60 and 75% of the ED patients matching the criteria for sepsis or septic shock did not receive a sepsis diagnosis at discharge, hampering the prompt start of appropriate treatments after hospital entry [11]. Often, personnel at the ED assign plausible diagnoses based on signs and symptoms, to be later confirmed after hospitalization. However, the early recognition of sepsis is a key aspect of clinical practice that can determine the prognosis of the patient. Up to a quarter of pre-shock patients initially evaluated in the ED may develop organ dysfunction [41], without being identified as cases of sepsis by any risk score [42]. For this reason, the identification of laboratory tests that can be used for the early assessment of sepsis has become a priority for emergency care.

The most updated International Consensus Definitions for Sepsis and Septic Shock recommended to use qSOFA to identify patients with sepsis in the ED [2]. However, there is no gold standard agreed in this context, where SIRS and organ failure are often caused by non-infectious diseases. On the other hand, the scoring systems adopted in the ED to predict sepsis are based on optimal thresholds (usually low) to minimise the percentage of false negatives [43, 44]. The adoption of MDW has been recommended to better recognise early sepsis and prevent clinical progression and death in the ED [31]. The qSOFA and NEWS (National Early Warning Score) have been frequently investigated as tools to screen patients with infection at risk of organ dysfunction and death [45]. Compared to SIRS, the use of qSOFA for sepsis has been found to be highly predictive for mortality, but less reliable as a screening tool for early recognition [46]. Moreover, the use of qSOFA showed high specificity for organ dysfunction, but low sensitivity to predict sepsis in the ED [44, 46, 47]. In fact, patients with a hidden infection may be present in the ED before showing the specific signs of sepsis detected by qSOFA (e.g., tachycardia, hypotension, and altered mental status) [48]. These findings justify our preference for SIRS criteria, as they are more sensitive, although less specific for sepsis detection.

In this study, we built a risk scoring algorithm showing moderately high accuracy, based on significant clinical characteristics and laboratory parameters. The advantage of this approach is that it can be directly applied to the next patient entering the ED, even through the aid of a simple pocket calculator. A value below threshold may be used to rule out sepsis. On the other hand, a value above threshold may not be as effective to rule in sepsis, due to its low PPV.

As expected, the scoring system assigns a very high weight to a SIRS ≥ 2. Hence, its application does not provide a substantial advantage over SIRS for this category of patients. On the other hand, the risk score may be most useful for those presenting with SIRS below 2, for which the diagnosis of sepsis is still uncertain [2].

A substantial barrier for the continuous update of this method is the limited interoperability of health databases in most European contexts, which can make the process of data acquisition particularly burdensome. Improving the digitalisation and standardisation of health information within and between hospitals in a privacy enhanced manner [49–52] can be paramount to strengthen the preparedness of health systems following the COVID-19 pandemic and to promote the adoption of research methods in clinical practice [53–55].

Finally, several strengths and limitations of our study are worth to be outlined.

Firstly, given the absence of an agreed gold standard for the diagnosis of sepsis, we used Sepsis-2 criteria, which may have led to a certain degree of misclassification, hampering the accuracy of clinical variables and biomarkers. However, we tried to limit this risk by assigning all uncertain cases to specific categories, after reviewing and discussing them carefully.

Secondly, host-related factors, e.g. HIV infection, ≥ 2 week steroid or other immune suppressive treatments, e.g. Hydroxyurea or Methotrexate, and Alcoholism may have influenced immune cell volumetric evaluation and other cell blood examination, potentially affecting the outcome prediction of these biomarkers. Nevertheless, we included all patients, after considering that given that all these preexisting conditions are present only in a small percentage of patients admitted to the ED, they would potentially affect only a small fraction of cases [22].

Thirdly, some study variables may have not been recorded accurately, e.g. smoking status and comorbidities, which were collected ex post through the examination of the medical record at ED. However, this problem may be limited by assuming that inaccurate measurements are randomly distributed across the study sample.

Fourthly, we enrolled a small number of patients due to the monocentric design of the study. Multi-centric investigations will be needed to improve the stability and reliability of the results.

Finally, the stepwise selection process may have potentially introduced selection bias in the identification of the final model. However, the backward elimination method that we have applied is generally considered more robust [56]. Moreover, we have used three different thresholds to mitigate the effect of the method and applied cross-validation to evaluate the accuracy of the results. The final estimates of the regression coefficients were used in two different ways for the prediction score algorithm, achieving the same predictive accuracy. However, the proposed algorithm appeared more directly intuitive for clinical readers, as they are used to relate to odds ratios for the interpretation of risk estimates.

## Conclusion

In this study, we have identified clinical and laboratory parameters associated with a diagnosis of sepsis in a population admitted to an ED of a large regional hospital.

Among clinical and laboratory variables, a value of MDW ≥ 22 was associated with an almost three-fold increased risk of sepsis. Risk scores derived from multivariate models showed moderate to high predictive accuracy in flagging subjects with sepsis, based upon the early evaluation of personal characteristics at the ED.

Our results suggest that incorporating MDW in clinical decision making may improve the early identification of sepsis in the ED, with limited additional effort over the existing standard procedures. Further multicentric studies are needed to confirm our findings.

## Data Availability

The datasets used and/or analysed during the current study are available from the corresponding author on reasonable request.

## Abbreviations

MDW: Monocyte Distribution Width
SIRS: Systemic inflammatory response syndrome
qSOFA: Quick sequential organ failure assessment
SSC: Surviving sepsis campaign
ICU: Intensive care units
ED: Emergency Department
PGH: Pescara General Hospital
AMI: Acute myocardial infarction
TIA: Transient ischemic attack
CVD: Cardiovascular diseases
COPD: Chronic obstructive pulmonary disease
CRP: C-reactive protein
WBC: White blood cells
ESRD: End-stage renal disease
AIDS: Acquired Immune Deficiency Syndrome
CBC: Complete blood count
RBC: Red blood cells
HGB: Haemoglobin
HCT: Hematocrit
MCV: Mean cell volume
MCHC: Mean cell hemoglobin concentration
MPV: Mean platelet volume
PDW: Platelet cell distribution width
RDW: Red cell distribution width
PCT: Procalcitonin
PPV: Positive predictive value
NPV: Negative predictive value
AUC: Area under the curve
OR: Odds ratios
95%CI: 95% Confidence interval
